# Supplementary material for: MetaRibo-Seq measures translation in microbiomes
Source: Nat Commun. 2020 Jun 29;11:3268. doi: 10.1038/s41467-020-17081-z (PMC7324362; doi:10.1038/s41467-020-17081-z)
Supplement: Supplementary file 10 — Supplementary Data 7 [file 41467_2020_17081_MOESM10_ESM.zip › File2/Confidence_High_Taxonomy/387905_out.krona.html]

Javascript must be enabled to view this page.

members
magnitude
magnitudeUnassigned
count
unassigned
taxon
rank

387905\_out

30


SRS014613\_contig\_number\_contig-100\_27904.69823SRS017701\_contig\_number\_contig-100\_9242.99171SRS149784\_contig\_number\_17878
3

superkingdom
3
2759

kingdom
4751
3

phylum
1
1913637

214504
1
subphylum

class
1
214506

1
36750
order

family
36751
1


SRS015782\_contig\_number\_contig-100\_1706.241513
genus
1
1129544

2
451864
subkingdom

phylum
5204
2

subphylum
2
29000

class
2
162481

231213
2
order

1799696
2
family

genus
2
5533

29898
2

SRS077024\_contig\_number\_986SRS149784\_contig\_number\_17129
species

2
24
superkingdom

1224
1
phylum

1236
1
class

1
135613
order

family
72276
1

genus
1
106633


SRS077231\_contig\_number\_contig-100\_2146.131869
species
108003
1

23
1239
phylum

5
1897031

SRS015782\_contig\_number\_46899SRS019068\_contig\_number\_37841SRS019445\_contig\_number\_26337SRS075773\_contig\_number\_36906SRS148196\_contig\_number\_25678
species


SRS020394\_contig\_number\_8978SRS064276\_contig\_number\_31704SRS104400\_contig\_number\_10426SRS143342\_contig\_number\_10121SRS143895\_contig\_number\_1718SRS147346\_contig\_number\_56902SRS148159\_contig\_number\_31610
species
7
1263007

11
186801
class

order
11
186802

family
216572
9

1952970
4
species

SRS016203\_contig\_number\_contig-100\_13.78718SRS063985\_contig\_number\_27885SRS077392\_contig\_number\_40249SRS104975\_contig\_number\_14999

459786
2
genus


SRS015431\_contig\_number\_62753SRS143342\_contig\_number\_15097
species
2
1945593


SRS1041032\_contig\_number\_341SRS1041039\_contig\_number\_29632SRS1041137\_contig\_number\_contig-100\_24.175445
species
3
1952972

1
1897049

SRS042966\_contig\_number\_18645
species

1897045
1
species

SRS056259\_contig\_number\_31339
